# Evaluating the diagnostic efficacy of whole-body MRI versus ^123^I-*m*IBG/^131^I-*m*IBG imaging in metastatic pheochromocytoma and paraganglioma

**DOI:** 10.1038/s41598-024-64607-2

**Published:** 2024-06-15

**Authors:** Hiroshi Mori, Hiroshi Wakabayashi, Shintaro Saito, Kenichi Nakajima, Kotaro Yoshida, Tomo Hiromasa, Seigo Kinuya

**Affiliations:** 1 Department of Nuclear Medicine, Kanazawa University Hospital, 13-1 Takara-machi, Kanazawa, Ishikawa 920-8641 Japan; 2 Department of Functional Imaging and Artificial Intelligence, Graduate School of Advanced Preventive Medical Sciences, Kanazawa University, Kanazawa, Japan; 3 Department of Radiology, Kanazawa University Hospital, Kanazawa, Japan; 4 Department of Nuclear Medicine, Faculty of Medicine, Institute of Medical, Pharmaceutical and Health Sciences, Kanazawa University, Kanazawa, Japan

**Keywords:** Cancer, Oncology

## Abstract

This study aimed to compare tumor lesion detectability and diagnostic accuracy of whole-body magnetic resonance imaging (WB-MRI) and radioiodine-labeled meta-iodo-benzylguanidine (*m*IBG) imaging techniques in patients with metastatic pheochromocytoma and paraganglioma (PPGL). This retrospective study included 13 patients had pheochromocytoma and 5 had paraganglioma, who were all suspected of having metastatic tumors. Each patient underwent WB-MRI and ^123^I-*m*IBG as a pretreatment screening for ^131^I-*m*IBG therapy. Two expert reviewers evaluated WB-MRI, ^123^I-*m*IBG images, and post-therapy ^131^I-*m*IBG images for the presence of metastatic lesions in the lungs, bones, liver, lymph nodes, and other organs. Diagnostic measures for detecting metastatic lesions, including sensitivity, specificity, accuracy, positive predictive value (PPV), negative predictive value (NPV), and receiver operating characteristics (ROC)—area under the curve (AUC), were calculated for each imaging technique. We analyzed WB-MRI images for detecting metastatic lesions, which demonstrated sensitivity, specificity, accuracy, PPV, NPV, and AUC of 82%, 97%, 90%, 96%, 86%, and 0.92, respectively. These values were 83%, 95%, 89%, 94%, 86%, and 0.90 in ^123^I-*m*IBG images and 85%, 92%, 89%, 91%, 87%, and 0.91 in post-therapy ^131^I-*m*IBG images, respectively. Our results reveal the comparable diagnostic accuracy of WB-MRI to one of the *m*IBG images.

## Introduction

Pheochromocytoma and paraganglioma (PPGL) are rare chromaffin cell-derived tumors that originate from the adrenal medulla or extra-adrenal paraganglia^1^. These tumor cells express the cell membrane norepinephrine transporter (NET), also known as uptake-1, and secrete catecholamine. *Meta*-iodo-benzylguanidine (*m*IBG) is a structural analog of norepinephrine, and it is actively taken up by PPGL cells via NET. Whole-body scans and single-photon emission computed tomography/X-ray computed tomography (SPECT/CT) with ^123^I-*m*IBG scintigraphy are widely available and used to assess the spread of *m*IBG-avid metastases and identify suitability for ^131^I-*m*IBG therapy in patients with PPGL^2^.

Since the 1980s, ^131^I-*m*IBG therapy has been widely used as a targeted radionuclide therapy in patients with PPGLs and *m*IBG-avid lesions. Moreover, post-therapeutic imaging following ^131^I-*m*IBG therapy demonstrated enhanced metastatic lesion detectability compared to diagnostic ^123^I-*m*IBG scintigraphy^3,4^. Further, ^131^I-*m*IBG serves as a therapeutic and diagnostic agent for PPGL in individuals with *m*IBG-avid metastases.

The current international guidelines recommend whole-body magnetic resonance imaging (WB-MRI) for detecting the presence of different cancer types^5^. Its effectiveness in detecting cancers and cancer-predisposition syndromes is well-documented, and it has numerous applications in clinical practice^5,6^. WB-MRI is recommended as a cancer screening image tool for individuals with a genetic predisposition for PPGL^7,8^, but its efficacy in detecting metastases in these patients and in comparison with ^123^I-*m*IBG and ^131^I-*m*IBG imaging techniques remains unknown. This study evaluated lesion detectability and the diagnostic characteristics of WB-MRI compared with ^123^I-*m*IBG and ^131^I-*m*IBG post-therapy in patients with metastatic PPGL.

## Materials and methods

### Study participants

The study included 18 patients, with data obtained from19 studies (a single hospital, Kanazawa University Hospital, Japan), who underwent WB-MRI and ^123^I-*m*IBG within one month as pretreatment to screen for ^131^I-*m*IBG therapy eligibility at Kanazawa University Hospital (Japan), from August 2019 to January 2022. All the patients underwent surgical removal of primary tumor lesions and histologically confirmed PPGL. The average age of the patients was 53.5 years old (average age: 53.5 ± 16.1 years), 12 patients were male (male/female ratio: 12/6), and 13 had pheochromocytoma whereas 5 had paraganglioma. All the patients were suspected of having metastatic tumor spread (Table 1). One patient (#6) was found to have positive mutations in the succinate dehydrogenase subunit B (SDHB) gene, but the status of other patients remains unclear. Family history could not be confirmed for any of the cases. Two patients did not undergo ^131^I-*m*IBG therapy due to progressive or stable disease. One patient underwent WB-MRI with ^123^I-*m*IBG imaging and ^131^I-*m*IBG therapy twice.

**Table 1:** Patient characteristics. Patient profiles (note: case No. 5 is the same as No. 4), the location of primary lesions, pathological diagnoses, and the presence of metastases in the lung, bone, liver, lymph nodes, and other areas.

Case no.	Age (years)	Sex	Primary lesion	Pathological diagnosis	Sites of metastasis				
					Lung	Bone	Liver	Lymph node	Others
1	46	F	Left adrenal grand	Pheochromocytoma	++		+		
2	51	F	Right adrenal grand	Pheochromocytoma	++	++		++	
3	36	M	Retroperitoneum	Paraganglioma	+	++			
4	67	F	Left adrenal grand	Pheochromocytoma	+	++			++
5 (same as #4)	68	F	Left adrenal grand	Pheochromocytoma	+	++			++
6	80	M	Left adrenal grand	Pheochromocytoma		+			+
7	65	M	Left adrenal grand	Pheochromocytoma	++	++	+		
8	44	M	Right adrenal grand	Pheochromocytoma		++			
9	51	M	Right adrenal grand	Pheochromocytoma		+		+	
10	56	M	Unknown	Paraganglioma		+		+	+
11	22	M	Left adrenal grand	Pheochromocytoma	+ +	++	+		
12	62	M	Urinary bladder	Pheochromocytoma	+	++			
13	65	M	Retroperitoneum	Paraganglioma				+	
14	45	F	Retroperitoneum	Paraganglioma		++	+		+
15	71	M	Retroperitoneum	Paraganglioma		+			+
16	72	M	Right adrenal grand	Pheochromocytoma	+	+		+	
17	53	F	Right adrenal grand	Pheochromocytoma		+		+	++
18	28	M	Left adrenal grand	Pheochromocytoma	+	++			
19	34	M	Left adrenal grand	Pheochromocytoma				+	+

F: female, M: male, +: one or up to five metastases, ++: more than 5 metastases.

### Image review and analysis

Two experienced reviewers (with 10 and 12 years of experience in imaging), blinded to the diagnosis, evaluated the presence of metastatic lesions in the lungs, bones, liver, lymph nodes, and other organs on images taken using WB-MRI, ^123^I-*m*IBG, and ^131^I-*m*IBG. Imaging modalities and acquisition characteristics include WB-MRI, WB-diffusion weighted image, apparent diffusion coefficient map, T1-weighted image, T2-weighted image, short inversion time inversion recovery weighted image; ^123^I-*m*IBG images, whole-body scan and SPECT/CT images 24 h after ^123^I-*m*IBG injection (PDR Pharma Co., Ltd, 222 MBq/person), post-therapy ^131^I-*m*IBG images, whole-body scan and SPECT/CT images 72–96 h after ^131^I-*m*IBG injection (POLATOM, Otwock, Poland or PDR Pharma Co., Ltd, 5550–7400 MBq/person). Lesions on images were classified as “positive” when one or more metastatic lesions and as “negative” when no metastatic lesions were identified (Table 2). A third independent expert reviewer (16 years of experience) who considered and combined in the analysis all imaging modalities tested (^123^I-*m*IBG image, post-therapy ^131^I-*m*IBG image, WB-MRI, plain/contrast-enhanced CT images, ^18^F-FDG-PET/CT images) and clinical information, established the gold standard.

**Table 2:** Category of number of metastases. Other locations of metastases: pleural dissemination (2 cases), retroperitoneal and pelvic dissemination (1 case), pleural invasion (1 case), local recurrence of retroperitoneal paraganglioma (1 case), posterior mediastinum (1 case), and peritoneal dissemination (2 cases).

No. of metastases	Lung		Bone		Liver		Lymph nodes		Others	
> 5	4	21%	10	53%	0	0%	1	5%	3	16%
1–5	6	32%	6	32%	4	21%	6	32%	5	26%
0	9	47%	3	16%	15	79%	12	63%	11	58%
Total cases	19	100%	19	100%	19	100%	19	100%	19	100%

### Diagnostic measures

We calculated the following diagnostic measures to evaluate the presence of metastatic tumor lesions in the different organs considered: sensitivity, specificity, accuracy, positive predictive value (PPV), negative predictive value (NPV), and receiver operating characteristics (ROC)—area under the curve (AUC). AUC was calculated for each of the organs considered (lung, bone, liver, lymph node, and other organs). The diagnostic tests were compared using ROC—AUC and DeLong’s tests. Cohen’s kappa statistics were used to evaluate reviewers’ consistency.

### Imaging protocol

A 1.5T MRI system (Philips Healthcare, Best, The Netherlands), equipped with a 20-channel head-neck coil and a 32-channel dS torso coil was used to examine all patients. The WB-MRI protocol developed in this study included several MR sequences, starting with a WB-diffusion-weighted image in a coronal plane. The WB-diffusion-weighted image sequence utilized a repetition time/time to echo of 6800/70 ms, a field of view set at 320 × 480 mm, and a 96 × 140 matrix size. The slice thickness was 5.0 mm, and inversion recovery with short-time inversion recovery (STIR) was used for fat suppression. Additionally, images were acquired with two b-factors of 0 and 800 s/mm^2^. Other sequences within the WB-MRI scan protocol, such as STIR TSE, T1W TSE, In/Out GRE, and T2W TSE, were acquired in different orientations, each with specific imaging parameters. The coronal WB-diffusion-weighted images were used to reconstruct three-dimensional maximum intensity projection (3D-MIP) images.

Anterior and posterior whole-body planar images and SPECT/CT images were acquired using a dual-head gamma camera (Symbia Intevo Bold; hybrid SPECT/CT scanner, Siemens Medical Solutions USA Inc., Hoffman Estates, IL, USA) after obtaining WB-MRI images. A low-medium energy general-purpose collimator was used for the ^123^I-*m*IBG imaging. Whole-body scintigraphy using ^123^I-*m*IBG was conducted with a matrix size of 256 × 1024 and a scan speed of 15 cm/min. The energy window was set to 159 keV, with a variance of ± 7.5%, optimized for ^123^I isotope detection. The SPECT/CT imaging in the ^123^I-*m*IBG protocol used a matrix size of 128 × 128, with each pixel measuring 4.8 mm. The imaging process involved 60 views (30 steps) at 20 s/step, with a total of 12.5 min/bed, evenly split across two detectors. Dedicated processing equipment (syngo Acquisition Workplace, Siemens Medical Solutions USA Inc., Hoffman Estates, IL, USA) was used to reconstruct the SPECT images.

Similarly to the ^123^I-*m*IBG imaging protocol, a high-energy collimator was used for ^131^I-*m*IBG imaging. The protocol for ^131^I-*m*IBG whole-body scintigraphy used a matrix size of 256 × 1024 with a scan speed of 20 cm/min. The energy window was specifically configured at 364 keV with a 10% variance. A 128 × 128 matrix size, with a pixel size of 4.8 mm, involving 60 views (30 steps) at 15 s/step, resulting in 7.5 min/bed of imaging, equally divided between two detectors, was used for the SPECT/CT imaging under the ^131^I-*m*IBG protocol. The SPECT images were reconstructed using dedicated processing equipment (syngo Acquisition Workplace, Siemens Medical Solutions USA Inc., Hoffman Estates, IL, USA).

### Statistical analysis

The diagnostic tests were compared using ROC—AUC and DeLong’s tests. Cohen’s kappa statistics were used to evaluate reviewers’ consistency. Statistical analyses were performed using EZR software (ver.1.60)^9^ and JMP software (ver. 13.2.0). A *P*-value of < 0.05 indicated statistically significant.

### Ethical approval and consent to participate

This study was conducted with ethical considerations in accordance with the Declaration of Helsinki and the ethical guidelines for biomedical research involving human subjects. The present study was approved by the ethics committee of Kanazawa University (Kanazawa University Medical Ethics Review Committee, Approval No. 2023-086 [114337]). Due to this study’s retrospective nature, written informed consent from each patient was waived by the Kanazawa University Medical Ethics Review Committee.

## Results

### Detectability of metastatic lesions using WB-MRI and *m*IBG imaging

We calculated the sensitivity, specificity, accuracy, PPV, NPV, and AUC for detecting metastatic lesions using WB-MRI, ^123^I-*m*IBG, and post-therapy ^131^I-*m*IBG images (Table 3). The results were 82%, 97%, 90%, 96%, 86%, and 0.92 for WB-MRI, 83%, 95%, 89%, 94%, 86%, and 0.90 for ^123^I-*m*IBG images, and 85%, 92%, 89%, 91%, 87%, and 0.91 for post-therapy ^131^I-*m*IBG images, respectively. The AUC did not significantly differ across the imaging modalities tested, with *P*-values of 0.55 and 0.78 for WB-MRI versus ^123^I* m*IBG and ^131^I-*m*IBG, respectively. The kappa coefficients for WB-MRI, ^123^I-*m*IBG, and post-therapy ^131^I-*m*IBG images were comparable when we evaluated reviewers’ agreements, with values of 0.87, 0.94, and 0.88, respectively. Therefore, we concluded that WB-MRI images demonstrated lesion detectability comparable to ^123^I-*m*IBG and post-therapy ^131^I-*m*IBG images in patients with PPGL (Table 3).

**Table 3:** Diagnostic characteristics of each modality. This table presents the sensitivity, specificity, accuracy, PPV, NPV, AUC, and p-value of 123/131I-mIBG images and WB-MRI in detecting metastases of each organ from PPGL, k-value indicating inter-reviewer agreement between reviewer 1 and reviewer 2.

	Sensitivity (%)	Specificity (%)	Accuracy (%)	PPV (%)	NPV (%)	k-value	AUC	p-value
Total								
WB-MRI	82	97	90	96	86	0.87	0.92	N/A
^123^I-*m*IBG	83	95	89	94	86	0.94	0.90	0.55
^131^I-*m*IBG	85	92	89	91	87	0.88	0.91	0.78
Lung								
WB-MRI	65	94	79	93	71	0.78	0.80	N/A
^123^I-*m*IBG	80	100	89	100	82	1.00	0.89	0.08
^131^I-*m*IBG	78	100	88	100	80	1.00	0.89	0.08
Bone								
WB-MRI	94	100	95	100	75	1.00	0.96	N/A
^123^I-*m*IBG	91	67	87	94	57	0.83	0.80	0.11
^131^I-*m*IBG	93	100	84	100	75	1.00	0.96	1.00
Liver								
WB-MRI	75	100	95	100	94	1.00	0.88	N/A
^123^I-*m*IBG	50	100	89	100	88	1.00	0.75	0.13
^131^I-*m*IBG	63	96	88	83	89	1.00	0.77	0.39
Lymph node								
WB-MRI	86	96	92	92	92	0.66	0.91	N/A
^123^I-*m*IBG	86	96	92	92	92	0.88	0.91	1.00
^131^I-*m*IBG	83	95	91	91	91	0.87	0.89	0.32
Others								
WB-MRI	94	100	97	100	96	0.89	0.97	N/A
^123^I-*m*IBG	94	100	97	100	96	0.89	0.97	1.00
^131^I-*m*IBG	94	100	97	100	95	0.88	0.97	1.00

PPV: positive predictive value, NPV: negative predictive value, AUC: area under the curve, N/A: not applicable.

### Detectability of metastatic lesions in each organ using WB-MRI and *m*IBG imaging

**Figure 1:**
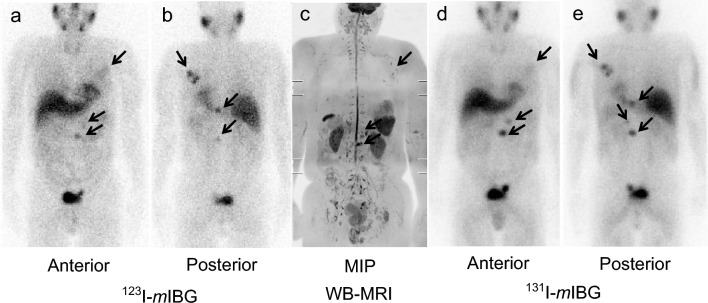
A case of multiple bone metastases (patient No. 6). A male patient with multiple bone metastases of pheochromocytoma (left scapula, thoracic, and lumbar vertebra, as indicated by black arrows). ^123/131^I-*m*IBG images detected more lesions compared with WB-MRI. Lumbar vertebral metastases were detected clearly by ^131^I-*m*IBG images. (**a,b**) ^123^I-*m*IBG scintigrams (anterior and posterior). (**c**) The MIP of WB-MRI. (**d,e**) Post-therapy ^131^I-*m*IBG scintigram (anterior and posterior). *mIBG meta*-iodo-benzylguanidine, *MIP* maximum intensity projection, *WB-MRI* whole-body magnetic resonance image.

**Figure 2:**
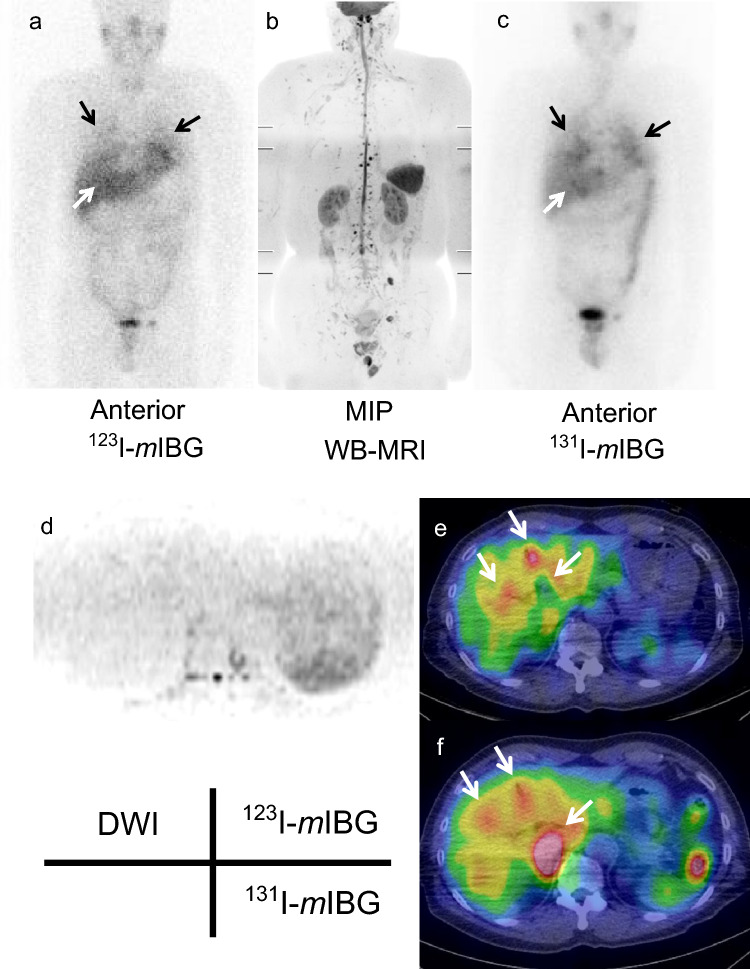
A case of multiple lung metastases with confusing liver accumulation in *m*IBG images (patient No. 7). A male patient in his 60s with multiple metastases of pheochromocytoma (lung and pelvis). Post-therapy ^131^I-*m*IBG image clearly detected multiple lung metastases (black arrows) compared with the ^123^I-*m*IBG image and WB-MRI. Conversely, *m*IBG images were confusing between liver metastases and physiologic accumulation (white arrows). (**a**) ^123^I-*m*IBG scintigram (anterior). (**b**) The MIP of WB-MRI. (**c**) Post-therapy ^131^I-*m*IBG scintigram (anterior). (**d**) DWI (axial). (**e**) ^123^I-*m*IBG SPECT/CT (axial). (**f**) Post-therapy ^131^I-*m*IBG SPECT/CT (axial). *WB-MRI* whole-body magnetic resonance image, *MIP* maximum intensity projection, *DWI* diffusion-weighted image, *T2WI* T2 weighted image, *mIBG meta*-iodo-benzylguanidine, *SPECT/CT* single-photon emission computed tomography/computed tomography.

**Figure 3:**
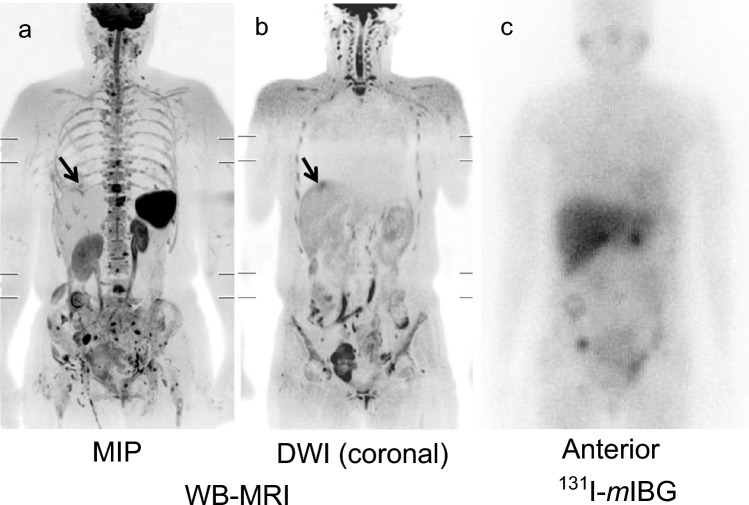
A case of liver metastasis that could only be detected by WB-MRI (patient No. 14). A female patient in her 40s with multiple metastases of paraganglioma (liver, vertebra, pelvis, peritoneum). Liver metastasis could only be detected by WB-MRI (arrows). Conversely, *m*IBG images could not detect metastasis due to physiologic liver accumulation. (**a,b**) The MIP of WB-MRI and DWI coronal image (WB-MRI). (**c**) Post-therapy ^131^I-*m*IBG scintigram (anterior). *WB-MRI* whole-body magnetic resonance image, *MIP* maximum intensity projection, *DWI* diffusion-weighted image, *mIBG meta*-iodo-benzylguanidine.

**Figure 4:**
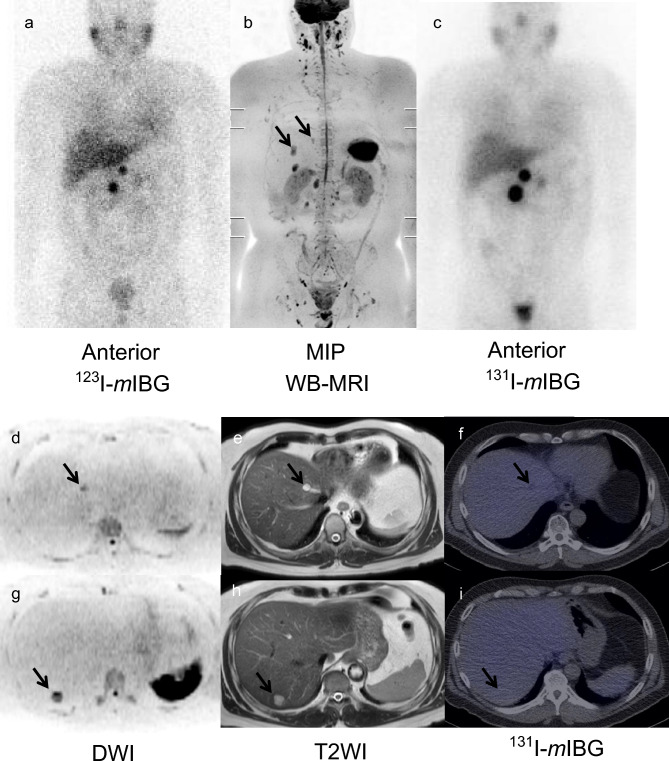
A case of WB-MRI confusing liver metastases with benign hepatic lesions (patient No. 9). A male patient in his 50s with multiple bone metastases of pheochromocytoma. Liver metastases, in addition to the metastases, were suspected by WB-MRI alone (arrows). Abnormal signal density and structures of DWI and T2WI revealed liver cysts, with no abnormal accumulation on post-therapy ^131^I-*m*IBG images. (**a**) ^123^I-*m*IBG scintigram (anterior). (**b**) The MIP of WB-MRI. (**c**) Post-therapy ^131^I-*m*IBG scintigram (anterior). (**d,g**) DWI (axial). (**e,h**) ^123^I-*m*IBG SPECT/CT (axial). (**f,i**) Post-therapy ^131^I-*m*IBG SPECT/CT (axial). *WB-MRI* whole-body magnetic resonance image, *MIP* maximum intensity projection, *DWI* diffusion-weighted image, *T2WI* T2 weighted image, *mIBG meta*-iodo-benzylguanidine.

The AUC for detecting metastatic lesions in the lungs, bones, liver, lymph nodes, and other organs were 0.80, 0.96, 0.88, 0.91, and 0.97 for WB-MRI images, 0.89, 0.80, 0.75, 0.91, and 0.97 for ^123^I-*m*IBG images, 0.89, 0.96, 0.77, 0.89, and 0.97 for post-therapy ^131^I-*m*IBG images, respectively. The AUCs and the *P*-values did not significantly differ among the tested imaging modalities. WB-MRI images in each of the body locations considered demonstrated lesion detectability comparable to ^123^I-*m*IBG and post-therapy ^131^I-*m*IBG images (Table 3). Either ^123^I/^131^I-*m*IBG or WB-MRI images distinctly detected some lesions (Figs. 1, 2). Notably, identifying or distinguishing physiological from abnormal findings in body regions adjacent to organs with high *m*IBG uptake on ^123/131^I-*m*IBG scintigraphy was possible using WB-MRI (Figs. 2, 3). Rib metastasis in patient No. 3 was detectable by both WB-MRI and *m*IBG images, but the extent of the metastatic lesions was more clearly depicted by WB-MRI images. In patient No. 13, multiple bone metastases (ribs, vertebrae, pelvic bones) were observed, with WB-MRI images providing a more detailed depiction. However, the use of WB-MRI did not allow for discrimination between liver metastases and benign liver lesions in one case (Fig. 4).

## Discussion

The diagnostic accuracy of WB-MRI was comparable to the one of *m*IBG images for all the lesions and organs considered in patients with metastatic PPGL in the study. WB-MRI was considered more valuable than ^123^I-*m*IBG scintigraphy because it detects lesions around organs with physiologic *m*IBG accumulation and allows clinical follow-up without exposing the patients to radiation. Additionally, the kappa coefficient revealed a substantial concordance among reviewers, indicating high reproducibility of the obtained results.

WB-MRI is currently an established screening tool for malignancies^10^, and international guidelines recommend it for screening patients with malignant tumors^5^. WB-MRI exhibits high diagnostic performances in identifying several tumor types, with 86% sensitivity for tumors, 80% sensitivity and 75% specificity for lymph node metastases, and 96% sensitivity and 82% specificity for metastatic disease determination^11^. WB-MRI demonstrated better screening performances for PPGL predisposition to biochemical tests. However, a comprehensive comparison with other imaging modalities was unaddressed. This study revealed that WB-MRI demonstrated a detection rate similar to that of *m*IBG imaging, which is currently used as a standard tool in Japan to assess tumor progression in patients with metastatic PPGL.

Our study used both WB-MRI and *m*IBG imaging techniques to assess the detectability of tumor lesions and explore the diagnostic performances specific to each of the five organs included in the analysis. We classified images as “positive” if they detected one or more lesions (Table 2). The detection in each of the organs was inconsistent, with no significant difference in detection performances for lesions with organ metastasis.

WB-MRI’s detectability in the liver demonstrated no significant differences compared to ^123^I-*m*IBG or ^131^I-*m*IBG scintigraphy. However, WB-MRI holds greater potential for detecting liver metastases than ^123^I-*m*IBG and ^131^I-*m*IBG scintigraphy due to the physiological accumulation of *m*IBG in the liver. Congruently, both ^123^I-*m*IBG and ^131^I-*m*IBG images demonstrated lower sensitivity in detecting liver tumor lesions. These results are consistent with previous studies reporting the superiority of diffusion-weighted whole-body imaging with background body signal suppression (DWIBS) over *m*IBG scintigraphy for detecting metastatic lesions^12^. The number of cases with liver metastasis was small which could potentially bias the results, but our study revealed that WB-MRI can be used as a tool to detect liver metastases.

In lung metastases assessment, WB-MRI exhibited low detectability compared to ^123^I-*m*IBG and ^131^I-*m*IBG images. Petralia et al.^13^ revealed the detection of false-negative when analyzing lung metastases of < 4 mm and Nicholas et al.^14^ reported a low detection rate with MRI imaging for pulmonary nodules of < 4 mm. Hence, further technical improvements are required before considering WB-MRI a viable alternative to CT in routine clinical lung assessments. The limited spatial resolution may have hindered WB-MRI detection of diffuse micro-metastases. Resolution concerns could also arise concerning ^123^I-*m*IBG and ^131^I-*m*IBG scintigraphy, but this was not an issue in our study given the good *m*IBG accumulation in the included patients.

The high physiological signal intensity had the potential drawback of hindering WB-MRI assessment in case of LN metastases, but it also has some advantages, as it can detect lesions around organs with physiologically high *m*IBG accumulation. Both WB-MRI and ^123^I-*m*IBG scintigraphy require the combination of physiological and pathological findings for proper detection. This study demonstrated that two reviewers revealed almost identical detection capabilities in identifying lesions with minimal or negligible knowledge of physiological features. The detectability and concordance of others did not significantly differ with physiological findings, including pleural dissemination, retroperitoneal and pelvic dissemination, pleural invasion, local recurrence of retroperitoneal paraganglioma, posterior mediastinum, and peritoneal dissemination in 2, 1, 1, 1, 1, and 2 cases, respectively.

Previous reports have revealed the superior diagnostic performance of post-therapy ^131^I-*m*IBG images in PPGL and neuroblastoma compared to ^123^I-*m*IBG images^3,15^. However, the diagnostic performance of both modalities, including whole-body and individual organ assessments, revealed comparable results in this study. The kappa coefficients of the reviewers for WB-MRI, ^123^I-*m*IBG, and post-therapy ^131^I-*m*IBG images exhibited similar concordance levels. This similarity in diagnostic performance may be associated with the specific criteria selected, where a lesion was considered “positive” if it was detectable across multiple lesions (Table 2).

This study included patients with *m*IBG-avid metastases in the therapy, but notably, several factors, including genetic information and tumor heterogeneity, influenced the varying detection rate of ^123^I-*m*IBG scintigraphy. Previous studies reported the diagnostic performance of ^123^I-*m*IBG in primary or metastatic PPGL, indicating sensitivity of 28–100% and specificity of 70–100%^2,16^.

Koopmans et al. reported that both ^111^In-pentetreotide and anatomical imaging (CT/MRI) showed a sensitivity of 93% in the detection of head and neck paraganglioma (HNPGL), with that of ^123^I-*m*IBG imaging being only 44%, suggesting that ^111^In-pentetreotide is a more suitable functional imaging tool for assessing HNPGL disease, particularly when there is high clinical suspicion and negative ^123^I-*m*IBG scintigraphy^17^. Fluorodeoxyglucose labeled with fluorine-18 (^18^F-FDG) is used to detect various tumors, and ^18^F-FDG PET has demonstrated high sensitivity (83%) for metastatic diseases of pheochromocytoma (PCC) and paraganglioma (PGL) in patients with mutations in the succinate dehydrogenase subunit B (SDHB) gene^18^, making it valuable for localizing metastatic PPGLs that are negative for *m*IBG^19^. It images glucose metabolism in tumors and has been reported to have no correlation with the uptake and storage of catecholamines^19^. However, ^18^F-FDG has limited specificity for qualitative diagnosis due to uptake in various tumors^18,20^. 3,4-dihydroxy-6-[^18^F]fluoro-L-phenylalanine (^18^F-DOPA) PET exhibits high sensitivity and specificity for detecting non-metastatic PCCs (both 100%)^21^, but sensitivity decreases in metastatic PCC and PGL diseases associated with SDHB mutations when compared to CT/MRI (20–45%)^18,22^. The loss of the norepinephrine transporter in dedifferentiated metastatic lesions may contribute to this reduced sensitivity^18^. Kroiss et al. reported the superiority of ^18^F-DOPA PET/CT over ^123^I-*m*IBG scintigraphy in ten patients with extra-adrenal PGL^23^. While ^18^F-DOPA PET and diagnostic CT detected all HNPGL, ^18^F-DOPA PET occasionally failed to detect bone metastases found by diagnostic CT, leading to an overall reduced sensitivity of 69.2% for malignant HNPGLs. Janssen et al. reported that the superiority of ^68^Ga-DOTA(0)-Tyr(3)-octreotate (^68^Ga-DOTATATE) PET/CT over ^18^F-FDG and ^18^F-FDOPA PET/CT in the localizing sporadic metastatic PPGLs^24^. Recently, studies have shown the advantages of ^18^F-*meta*-fluorobenzylguanidine (^18^F-*m*FBG) PET/CT over ^68^Ga-DOTATATE PET/CT in patients with metastatic PPGLs^25,26^. It is suggested that a combination of functional and anatomical imaging is necessary to improve tumor detection. Therefore, a combination of imaging evaluations, including *m*IBG scintigraphy, somatostatin receptor imaging, and PET tracer imaging, is necessary to prepare for nuclear medicine treatment in a clinical setting. This indicates that results may differ when *m*IBG-negative patients are included.

Future studies focusing on the *m*IBG-negative patient group are required to further consolidate data on detection rates. Conducting follow-up observations in *m*IBG-negative patients using radiation-free imaging techniques may be possible if a high detection rate similar to the one of WB-MRI could be achieved.

This study is not without limitations. First, the number of patients included was limited, but this reflects the rarity of PPGL as a disease. Hence, minor differences observed among the imaging modalities under evaluation may not have reached statistical significance. Second, not all the metastatic lesions that demonstrated positive findings in WB-MRI and *m*IBG images underwent pathological diagnosis. This may introduce the possibility of false-positive findings. Third, the assessment did not account for the precise number of metastases because this study focused on the presence and extent of metastases, and any abnormal *m*IBG accumulation was considered “positive”.

## Conclusion

This study revealed that WB-MRI and *m*IBG images demonstrated comparable diagnostic accuracy. However, we observed that WB-MRI holds some advantages as a technique for detecting lesions that are in proximity to organs characterized by physiological *m*IBG accumulation. Furthermore, WB-MRI offers a radiation-free alternative for follow-up examinations in patients with PPGL. WB-MRI may play a significant role in the long-term follow-up and treatment of patients with PPGL. Further research in the field is required to corroborate our findings and open new clinical management avenues for these patients, considering the retrospective study design.

## Data Availability

The data of this study are available from the corresponding author (HM) upon reasonable request.

## Abbreviations

CT: Computed tomography
MRI: Magnetic resonance image
SPECT/CT: Single-photon emission computed tomography/X-ray computed tomography
PPGL: Paraganglioma
*m*IBG: *Meta*-Iodo-benzylguanidine
WB-MRI: Whole-body magnetic resonance image
DWIBS: Diffusion-weighted whole-body imaging with background body signal suppression
PPV: Positive predictive value
NPV: Negative predictive value
ROC: Receiver operating characteristics
AUC: Area under the curve
FDG-PET/CT: Fluorodeoxyglucose positron emission tomography/computed tomography
NET: Norepinephrine transporter
SDHB: Succinate dehydrogenase subunit B
HNPGL: Head and neck paraganglioma
PCC: Pheochromocytoma
PGL: Paraganglioma
^18^F-DOPA: 3,4-Dihydroxy-6-[^18^F]fluoro-L-phenylalanine
^68^Ga-DOTATATE: ^68^Ga-DOTA(0)-Tyr(3)-octreotate
^18^F-*m*FBG: ^18^F-*meta*-fluorobenzylguanidine
