# Publisher Correction: Evaluating the diagnostic efficacy of whole-body MRI versus ^123^I-*m*IBG/^131^I-*m*IBG imaging in metastatic pheochromocytoma and paraganglioma

**DOI:** 10.1038/s41598-024-68300-2

**Published:** 2024-08-06

**Authors:** Hiroshi Mori, Hiroshi Wakabayashi, Shintaro Saito, Kenichi Nakajima, Kotaro Yoshida, Tomo Hiromasa, Seigo Kinuya

**Affiliations:** 1 Department of Nuclear Medicine, Kanazawa University Hospital, 13‑1 Takara‑machi, Kanazawa, Ishikawa 920‑8641 Japan; 2 Department of Functional Imaging and Artifcial Intelligence, Graduate School of Advanced Preventive Medical Sciences, Kanazawa University, Kanazawa, Japan; 3 Department of Radiology, Kanazawa University Hospital, Kanazawa, Japan; 4 Department of Nuclear Medicine, Faculty of Medicine, Institute of Medical, Pharmaceutical and Health Sciences, Kanazawa University, Kanazawa, Japan

Correction to: *Scientific Reports* 10.1038/s41598-024-64607-2, published online 15 June 2024

In the original version of this Article the captions of Table 1, Table 2 and Table 3 were erroneously omitted.

The original Article has been corrected.

